# Cultural significance of Lepidoptera in sub-Saharan Africa

**DOI:** 10.1186/s13002-019-0306-3

**Published:** 2019-06-13

**Authors:** Arnold van Huis

**Affiliations:** Laboratory of Entomology, Wageningen University & Research, P.O. Box 16, 6700 AA Wageningen, The Netherlands

**Keywords:** Art, Butterflies, Caterpillars, Entomophagy, Ethno-entomology, Ethno-medicine, Literature, Metamorphosis, Moths, Proverbs

## Abstract

**Background:**

The taxon Lepidoptera is one of the most widespread and recognisable insect orders with 160,000 species worldwide and with more than 20,000 species in Africa. Lepidoptera have a complete metamorphosis and the adults (butterflies and moths) are quite different from the larvae (caterpillars). The purpose of the study was to make an overview of how butterflies/moths and caterpillars are utilised, perceived and experienced in daily life across sub-Saharan Africa.

**Method:**

Ethno-entomological information on Lepidoptera in sub-Saharan Africa was collected by (1) interviews with more than 300 people from about 120 ethnic groups in 27 countries in the region; and (2) library studies in Africa, London, Paris and Leiden.

**Results:**

Often the interviewees indicated that people from his or her family or ethnic group did not know that caterpillars turn into butterflies and moths (metamorphosis). When known, metamorphosis may be used as a symbol for transformation, such as in female puberty or in literature regarding societal change. Vernacular names of the butterfly/moth in the Muslim world relate to religion or religious leaders. The names of the caterpillars often refer to the host plant or to their characteristics or appearance. Close to 100 caterpillar species are consumed as food. Wild silkworm species, such as *Borocera* spp. in Madagascar and *Anaphe* species in the rest of Africa, provide expensive textiles. Bagworms (Psychidae) are sometimes used as medicine. Ancestors may be associated with certain dark nocturnal moths, but these are also considered to be responsible for armyworms plagues. The appearance of butterflies/moths can be associated with seasons or serve as predictor of events. There are many proverbs, songs and stories related to butterflies and moths. Lepidoptera are also an inspiration in art expressions. In dance, the movements of caterpillars are used as examples, while certain cocoons are used as rattles.

**Conclusion:**

Lepidoptera are found very appealing because of the striking appearance of the adults, their dramatic metamorphosis and the provision of silk and nutritious food. Besides, they are an inspiration in art and literature.

## Background

Butterflies and moths make up the order of the Lepidoptera. The most prominent features of the Lepidoptera are the scales and the proboscis. The scales are flattened hairs that cover the body and the wings and are the source of large variety of colour patterns. The proboscis is tubular and is coiled under the head. By pumping liquid in the proboscis, it elongates. Members of the order of the Lepidoptera are holometabolous, which means they have a complete metamorphosis, a form of insect development which includes four life stages—an imago or adult (moth or butterfly), an embryo or egg, a larva (caterpillar) and a pupa or chrysalis, during which the adult structures are formed. The silken cocoons are spun by the larvae at maturity, although many species pupate without a cocoon. The taxon Lepidoptera is one of the most widespread and widely recognisable insect orders in the world. Worldwide, there may be more than 350,000 species. Approximately 160,000 species have been described, about 20,000 of which are from Africa [1]. The adults provide ecological services such as pollination and food in the food chain. However, for the larvae, the main food is living plant material and therefore they can be agricultural pests.

The taxon Lepidoptera consists of butterflies (suborder Rhopalocera) and moths (suborder Heterocera). Most species of the order Lepidoptera are moths with many super families; butterflies have only two super families. The larvae of both butterflies and moths are called caterpillars. The distinction between the two groups in the adult stage is anatomical (e.g. butterflies have club-antennae and moth varied-antennae) and behavioural (e.g. butterflies are often active by day, and moths by night). In my interviews, people were often not aware of the difference and for that reason, when not being able to track it down, I name in this article adults of both groups either ‘butterflies (/, and, or) moths’ or lepidopterans.

Two families featuring in this article will be highlighted. One are bagworms (family Psychidae) of which the larvae live in portable cases constructed from silk and plastered with symmetrically arranged fragments of leaves, sticks and thorns. Scalercio and Malaisse [2] provide an overview of edible bagworms in Africa. The females are wingless, legless and wormlike, and usually never leave the bag in which they pupate. Another group is the Saturniidae, the emperor or giant silk moths; they are medium sized to very large, broadly winged and have a highly variable colour pattern.

## Methods

The information was collected by reviewing the literature and by personal interviews. The interviews were conducted in the years 1995 and 2000 in Africa and concentrated on the traditional, nutritional and medical uses of arthropods and their products as well as on their role in religion, witchcraft, art, song, music, dance, children’s games, mythology and literature. A part of the results obtained in 1995 on insects has been published [3], as well as the part on edible insects over both years (1995 and 2000) in 2003 [4].

The total number of people interviewed was 304 from 27 different countries in sub-Saharan Africa of whom 22 were resource persons (experts without recorded ethnic affiliation) (Table 1). Of five other respondents, the ethnic group was unknown. The total number of ethnic groups was 121, excluding Zanzibar and Madagascar where the ethnicities were not recorded. Names of ethnic groups were checked, mostly in Wikipedia and the Joshua project [5].

**Table 1:** The number of respondents (*N*) per country and ethnic group

Country	Ethnic group—*N*	RES^a^	N
Benin	Bariba-1, Fon-4, Goun-1, Nagot-6, Popo-1, Tori-1		14
Burundi	Hutu-2		2
Burkina Faso	Mossi-4, Fula-1		5
Cameroon	(Bamileke-14, Bafia-1, Bakoko-1, Bakossie-1, Banen-1, Bani-Pahuin-1, Bassas-2, Beti-Eton-1, Beti-Ewondo-1, Bolous-1, Matha-1, Tikar-1, Wimboum-1, Yambassa-1	2	28
CAR^b^	Gbaya-1, Kari-1		2
DRC^c^	Mbochi-1, Teke-1		2
Chad	Arabe-1, Goulaye-2, Kanembou-1, Mbaye-2, Ngambaye-7, Sara-Kaba-1, Sara-Niellim-1, Tupuri-1, Wadai-1		17
Gambia	Jola-1, Mandinka-1		2
Guinee-Bissau	Balanta-1		1
Kenya	Kalenjin-1, Kamba-4, Kikuyu-2, Luo-4, Meru-1, Somalian- 1		13
Madagascar	–		24
Malawi	Chewa-1		1
Mali	Fula-1, Mande-Malinke-1, Mande-Mandinka-1, Sarakolé-1, Senufo-2, Songhay-3, Tuareg-1		10
Mozambique	Bitonga-1, Makua-1, Nchope-1, Shona-1, Tsonga -Rhonga-2, Tsonga- Shangana-1, Tsonga-Tswa-1		8
Namibia	Damara-1		1
Niger	Djerma-1, Hausa-9, Kanuri-1, Songhai-4	1	15
Nigeria	Ebibio-1, Ebira-1, Yoruba-15, Unknown-1		18
Rwanda	Kiga-Toro-1		1
Senegal	Bainuk-1, Diola-4, Fula-1, Halpulaar-2, Lebu-1, Serer-3, Wolof-5		17
South Africa	–	6	
Sudan	Dongolawi-1, Fula-1, Gaälien-3, Kambari-Abadi-1, Kawahla-1, Kuku-1, Mahas-1, Nubian-1, Nubian-Mahas-1, Rubatab-2, Tunyur-1, unknown- 4	5	18
Tanzania	Chaga-7, Digo-1, Iraqw-3, Iramba-1, Mwarusha-2, Pare-1, Rangi-1, Sukuma-2, Zanaki-1	1	19
Togo	Akebu-1, Ewe-5, Cotocoli-1, Kabye-1, Mina-1	3	9
Uganda	Acholi-1, Banyankole-1, Bunyoro-1, Busoga-1, Ganda-7, Langi-1, Luo-2, Nyoro-1		15
Zambia	Bemba-1, Ila-1, Lovale-1, Lozi-2, Lunda-1, Namwanga-2, Nyanja- Chewa-1, Tonga-10, Tumbuka-1	2	20
Zanzibar	–		9
Zimbabwe	Ndebele-1, Shona-9, Zezuru-1	2	11
Total number of resource persons and respondents		22	282

^a^Resource persons

^b^Central African Republic

^c^Democratic Republic of Congo

Most of the people interviewed were scientists or technicians trained in entomology. The interviewees were identified by visiting entomological groups of universities and (inter) national agricultural research institutes, plant protection services, museums and crop protection projects. The author tried to interview most of the staff of these organisations (often arranged by the responsible officers). The age of the persons interviewed varied between 25 and 65. Most of the respondents were male reflecting the gender composition of the organisations. A few times people were interviewed in villages who had no entomological background. This proved to be a challenge because of language and confusion about the insect species. Twenty-two of the respondents acted as resource persons on special subjects (for example experts on termites or insects as food or medicine) or had special positions (professors, heads of organisations, shamans, museum directors and priests). In these cases, the ethnic origin of the person who provided the information was not considered relevant.

The author used a list of issues to be covered in the interviews. A number of respondents got this list before the country was visited. Often, they questioned elders, grandparents, family members and acquaintances before my arrival. This information was then passed on to me. Respondent from rural areas had more information than those from urban areas. Considering the rapid urbanisation and the fact that older people often had to be consulted is a sign that this type of information is rapidly disappearing. Therefore, although the information was collected some 20 years ago, the information is not only valid, but probably more difficult to be obtained now.

Vernacular names and their meaning were double checked with the respondents and sometimes by literature search. The national libraries and university libraries in London and Paris, the library of the African Studies Centre in Leiden, the Netherlands and some libraries of the countries visited were consulted. The literature consulted was mainly of anthropological nature. Findings for a country or a certain tribe were only reported if information was received from more than one respondent, or if the information given during interviews was confirmed in the literature. The respondents’ countries and tribes are mentioned to specify the sources of information. They cannot be used for establishing correlations between ethnicity and information provided. The qualitative character of the information provided is emphasised.

## Results and discussion

### Nomenclature

Very often I was told that people do not know that butterflies and moths derive from caterpillars and thus are unaware of its complete metamorphosis (Burkina Faso: Fula; Chad: Mbaye, Ngambaye; Gambia: Jola; Kenya: Luo; Mali: Sarakolé, Mande-Madinka; Mozambique: Shangana; Niger: Hausa; Senegal: Serer; Sudan: Gaälien, Kuku, RES; Uganda: Acholi, Busoga, Bunyoro, Langi, Ganda).

In Senegal, the butterfly or moth is called in Halpulaar ‘déftèle-allah’ (paper of God) (HalPulaar) or in Fula ‘Bedèllel Allah’ (God’s fan). In Mali (Songhay), it is called ‘Alfaga-alfaga’, which means ‘marabout’. In Niger (Hausa), they call it ‘Malam batata’ (Malam = marabout; batata—pray), or in Chad (Kanembou) ‘Kouli malimi’ (insect = kouli; malimi = marabout). The name is explained by the wings moving up and down, like a Muslim who is praying. In Chad (Arabe), it was called ‘Aboune daguig’ (daguig is meal), probably referring to the wing scales which resemble powder. In Chad (Ngambaye), the name ‘Eube’ refers to instable, probably because of the erratic or spasmodic way of flying. In the same country (Sara-Niellim), the name of butterfly or moth is ‘borgue’, which means the soul of a deceased, reason that the animal is never touched. In Chad (Goulaye, RES), it is believed that the scales irritate the skin, another reason not to touch it. For caterpillars, it is known that they have urticating hairs which may give an (allergic) skin reaction, such as in Niger (Hausa) where a caterpillar *Amsacta* sp. is called ‘basusa’ which means ‘the one that causes scratching’.

The Mbaye, an ethnic group living in the south of Chad, classify the Lepidoptera (moths and butterflies) in three categories: (1) the coloured ones (Macrolepidoptera or Rhopalocera), (2) the less coloured moths often nocturnal (Microlepidoptera or Heterocera) and (3) the ones which are a pest of millet [6]. They seem to know very well the metamorphoses of lepidopterans. This classification is not made by the Chokwe and the Nkoya in the western part of Zambia. There they also call the insects butterflies/moths when they have a fluttering way of flying, such as net-winged insects (Neuroptera) or dragonflies (Odonata) ([7]; p. 179). These ethnic groups are aware of metamorphosis but are not able to recognise which butterfly or moth belongs to which caterpillar.

Butterflies or moths when setting on the ground, a branch or a flower, often open and close their wings. In Zambia, the Ngangala and Chokwe believe that they do so to fan and refresh themselves. However, the Nkoya (or Mashasha) in the same country believe that it is to free themselves of excrements, as they suffer from continuous constipation ([7]; p. 181).

**Fig. 1:**
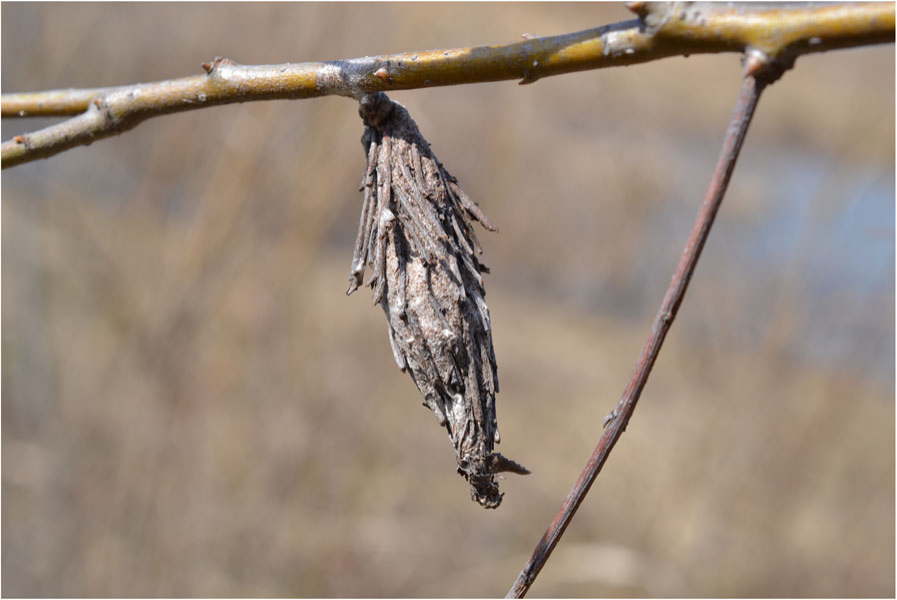
A typical bagworm. Source: https://commons.wikimedia.org/wiki/File:Bagworm_Moth_(Psychidae)_%22bag%22_(13315008883).jpg. Attribution: Andrew C [CC BY 2.0 (https://creativecommons.org/licenses/by/2.0)]

All my Bamileke respondents in Cameroon mentioned that the bagworm is called ‘Dak kué di Fock’ (Dak = ramasse; kué = bois; di = dormir; Fock = froid), which means ‘the one who collects wood, but sleep in the cold’ (Fig. 1). Reference is made to the fact that firewood is collected but that the caterpillar does not use it for fire. Also, in Uganda (Bunyoro, Ganda), the bagworm is called ‘collector of firewood’. Almost all my informants from the different ethnic groups in Cameroon indicated that the number of sticks covering the insect represents the age of the animal; see also Ittmann ([8]; p. 29). The different languages indicate this, e.g. ‘alan mimbou’ (count years) (Cameroon: Beti). In Cameroon, the bagworm *Eumeta cervine* is also called ‘the one that circumcises the penis’ by the Bafia [9] and in the Central African Republic (Gbaya) it is called the ‘pisseusse’ (the weak bladdered) ([10]; p. 279).

In Zambia (Chokwe and Ngangala), the local words used for bagworms denote girls undergoing initiation rites at puberty ([7]; pp. 166–172). These girls are kept isolated in especially erected huts covered with grass, like the shelter of the bagworm. This is also mentioned by the White Fathers [11] in Zambia (Bemba) where the bagworm ‘kantebele’ is used as a charm in the beginning of the cisunga ceremony, which is performed at the appearance of the first menstruation. The same authors mention that the term is also used metaphorically for a dandy (a man who dresses elegantly and fashionably) which is an allusion to the insect covering itself with many pieces of grass.

Malaisse ([12]; p. 208) reports that the vernacular name of edible caterpillars is often derived from the host they are collected from. For example, in Zimbabwe, a caterpillar is called ‘finamwenge’ in the bemba language; ‘fina’ means ‘coming from’ and ‘mwenge’ is the host, the rubber tree, *Diplorhynchus condylocarpon*. However, for the Gbaya in the DRC, this is only true when a caterpillar is confined to one host ([10]; pp. 281–282). Most of the 59 ethnospecies (about 40%) termed by the Gbaya are named after a characteristic, such as displacement, the sound they make, the urticating hairs and their use as medicine. Others are named according to their appearance (colour or stripes) or the period in which they are harvested.

### Insects as food and feed

A large number of caterpillar species are eaten in Africa [4, 13]. In 2017, a list of 97 species was compiled [14]. In central Africa, many caterpillar species are consumed ([10]; pp. 272–298, [12]; pp. 199–217, [15–18]; pp. 15–113). In southern Africa, one of most frequently eaten is the mopane caterpillar (*Gonimbrasia belina*) [19] with high nutritional value [20]. In West Africa (in particular Burkina Faso) and one country in central Africa (Chad), the caterpillar *Cirina butyrospermi* Vuillet and *Cirina forda* (Lep.: Saturniidae) from the karité tree (*Vitellaria paradoxa*) is a nutritious species (Burkina Faso: Mossi; Chad: Mbaye, Sara-Kaba; Mali: Fula, Senufo, Songhai) [21–23]. However, the Mossi and Fula migrants in the northern Sudanian zone in Burkina Faso do not eat it, probably due to cultural assimilation [24]. In Nigeria (Yoruba), *C*. *forda* (called ‘kanni’ or ‘munimuni’) caterpillars, collected from leaves or from pitfall traps around the bases of trees, are starved to eliminate the gut contents, boiled (or smoked) for a few hours, sun dried on mats and then cooked in a stew of vegetables [25]. In Nigeria, the caterpillar is twice as expensive as beef! The price is probably the reason that in Burkina Faso this species is even called ‘caviar of the savannas’ [26].

In the CAR (Gbaya), the caterpillars are sold alive, because there are no ways to conserve them. For this reason, the animals are collected very early in the morning.

Three *Anaphe* species are eaten: *Anaphe panda* (syn: *Anaphe infracta*), *Anaphe reticulata* and *Anaphe venata* [27–29](Lep.: Thaumetopoeidae) of which the larval stage are called processionary caterpillars, because they move in columns in search of food. These edible caterpillars were often mentioned in Cameroon (Bafia, Bamileke, Bani-Pahuin, Beti-Ewondo, Matha) and Nigeria (Yoruba). The first species has been farmed by feeding them with leaves of the tree *Bridelia micrantha* [29]. The second species also has *B*. *micrantha* as host tree but also other tree species. *Anaphe venata* lives on the timber tree abachi (*Triplochiton scheroxylon*) and cola trees. The local population often removes the skin-irritating hairs by fire (Cameroon: Bani-Pahuin, Beti-Ewondo, Matha). The caterpillars can be cooked fresh, fried or powdered for storage [30].

When a person from the Yansi in the Democratic Republic of Congo finds edible caterpillars in a tree, he/she claims the tree as being his/her property by marking it and he/she alone is allowed to harvest the caterpillars from this tree ([18]; p. 21); this is also mentioned by Latham ([16]; p. 40) for the 25-m-high tree *Macaranga monandra* in the DRC.

In Asia, it is very well-known that the pupae of the domesticated silkworm, *Bombyx mori* (Lep.: Bombycidae), are eaten. Apparently in Madagascar, also the pupae of the wild silkworm, *Borocera cajani* (Lep.: Lasiocampidae), are eaten ([31]; pp. 55–67).

There may be taboos involved. For example in the Ntomba tribe (Democratic Republic of Congo), the eating of certain caterpillars, probably *Acraea* sp. (Lep.: Nymphalidae), are not allowed to be eaten by twins [32].

*Cirina butyrospermi* has also been considered as an alternative of fishmeal to feed African sharptooth catfish [33, 34], while for chickens *A*. *panda* [35] and *Cirina forda* have been proposed [36, 37]. One respondent from Togo (Cotocoli) indicated that small butterflies/moths are used for fishing. The flapping of the wings above the water surface attracts fish.

### Silk

**Fig. 2:**
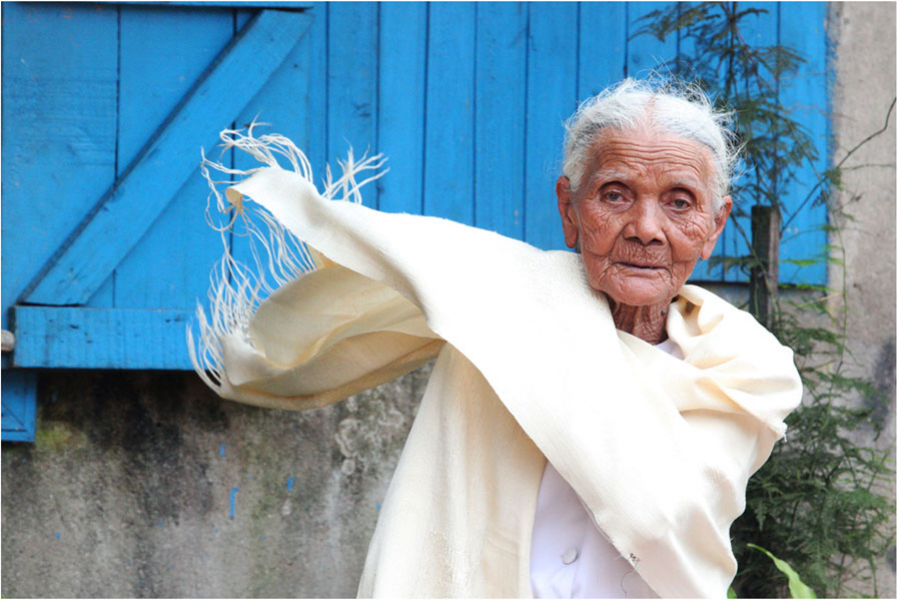
Elder Merina woman with a white lamba akotofahana in Madagascar. Source: https://commons.wikimedia.org/wiki/File:0006_Madagascar_(5564873899).jpg. Attribution: Steve Evans from Citizen of the World [CC BY 2.0 (https://creativecommons.org/licenses/by/2.0)]

A wild silkworm, i.e. *Borocera madagascariensis* (Lep.: Lasiocampidae), in the central highlands of Madagascar occurs mainly on tapia (*Uapaca bojeri*) trees [38]. In the last larval stage, the caterpillars spin the cocoon which they attach to branches of trees, before pupating. These pupae are considered a delicacy and are fried in oil. The cocoons are traded and a saying is ‘Bory manan-tongrota hoatra ny landy’, which means the cocoons have no limbs but have feet, because the cocoons are traded over long distances ([39]; p. 191). The amount of silk (‘landibe’) that can be harvested is less than that from the common silkworm *B*. *mori*. The small yield as well as the difficulty of harvesting because of the projecting urticant hairs, which can easily penetrate the skin causing infections, makes the silk very expensive in Madagascar (a piece of a few meter takes one and half month to make it and cost easily 100–200 US$). The caterpillar was semi-domesticated up to the 1940s [40]. The silken cloth was only worn by people of royalty ([41]; p. 552). Nowadays, only rich women wear this silk as ‘lamba landy’ on very special occasions. De ‘lamba’ is worn around the shoulders and draped around the head with the end pointed backwards (Fig. 2).

Deceased important persons are wrapped in ‘lamba mena’ cloth made from ‘landibe’ silk ([41]; p. 552). The ethnic group Merina and later the Betsileo, however, bury their deceased twice during ‘famadihana’ which means ‘the turning of the bones’ ([42]; p. 33). This second time is 5 years after the initial burial and this time the deceased is wrapped in ‘lamba landy’, a lighter type of cloth, which is wrapped over the ‘lamba mena’. It should however not be too thin, according to the saying ‘Lamban angidina: ka tafiam-belona, tsy mahatafy; ifonosa-maty, tsy mahafono’; ‘lamba-angidini’ means literally ‘the cloth of a dragonfly’; it is too thin to wear as cloth and so also too thin to bury the dead ([39]; p. 80). In the chalk formations in the central highlands of Isalo, the permanent graves are often found in caves of steep mountain walls. The explanation given was that ‘the stealing of the precious silk is more difficult when the deceased was close to God’. The second burial is an expensive ceremony of several days and many guests are invited and many zebus (cows) are slaughtered. The ancestors are in this way honoured as they are the connection between God and humans. Ancestors are honoured and consulted by giving offers, often honey, on the graves. This is done when important decisions must be made such as the building of a house or marriage.

*Anaphe* caterpillars (such as *A. venata*) (Lep.: Notodontidae) mass together (about 300) and they make a communal cocoon, which in Nigeria (Yoruba) is processed into silk yarn and woven into special cloth called ‘sanyan’ or ‘aso oke’. These cloths are worn during special ceremonies such as funerals. Only rich people can afford them. Children use the cocoon nest as a purse (Cameroon: Bakossie), adults as a receptacle to conserve gun powder for the hunt ([16]; p. 22). The high quality of *A*. *panda* silk provides excellent opportunities for African countries. Mbahin et al. [43], who studied their occurrence on the tree *B*. *micrantha*, recommended the management of indigenous forests in a sustainable way in Africa as the occurrence of wild silk moths is declining due to deforestation and overconsumption.

### Medicine

A traditional healer from Togo told me that one should use seven species of butterflies to facilitate child delivery. You need to soak the herbs in water and the pregnant wife needs to bath herself on the road. The same informant told me that a bat and butterflies/moths with honey should be eaten against palpitations. The very large cocoon of *A*. *panda* is burned, and the fumes cure severe headaches (Uganda: Ganda, Nyoro).

Often bagworms are used as medicine and in witchcraft (Cameroon: Bamileke, Bani-Pahuin, Beti-Eton; Uganda: Ganda, Zambia: Bemba, Tonga). In Madagascar, it was mentioned as medicine against nocturnal enuresis in children. Similarly, Roulon-Doko ([10]; p. 279) reports this for the Gbaya in the DRC, explaining the name given to this insect ‘pisseusse’. Other ailments that can be cured by the bagworm are tonsil problems (Zambia: Bemba), iodine deficiency (Zambia: RES) and ear trouble (Zimbabwe: Shona). In Zambia, Silow ([7]; p. 171) mentioned that the Nkoya and Mbunda use bagworms for a number of health problems: hiccups, coughing, nose bleeding, haemorrhage (mouth; menstrual) and at childbirth, when the afterbirth is late. He also mentions that Nkoya women in Zambia smear the belly with bagworms’ ashes during difficult deliveries, remedies also used by doctors.

### Religion and superstition

In many cultures, the soul of a dead person is associated with a moth or butterfly, e.g. in ancient Greece the word ‘butterfly’ is ‘psyche’ relating to ‘soul’, thought to be the soul of the dead [44]. To quote these authors:

While the butterfly symbolises awe, the moth has become the unwilling symbol for that which is ugly and negative. Other symbols identified with moths – such as insanity, for example – are also responsible for the moth’s low esteem. However, the moth, attracted by the flame – just as the soul is by heavenly truth – burns itself in the flame, reflecting the trials that must be endured to eliminate the flesh before knowing the joys of the beyond.

Would there be a similar association in sub-Saharan Africa? In Sudan (Dongolawi, Gaälien, Nubian) and Rwanda (Kiga-Toro), naming a person a butterfly or moth means that he/she is a nasty quarrelling and unreliable person which insists so much that the person in the end will burn him/herself.

**Fig. 3:**
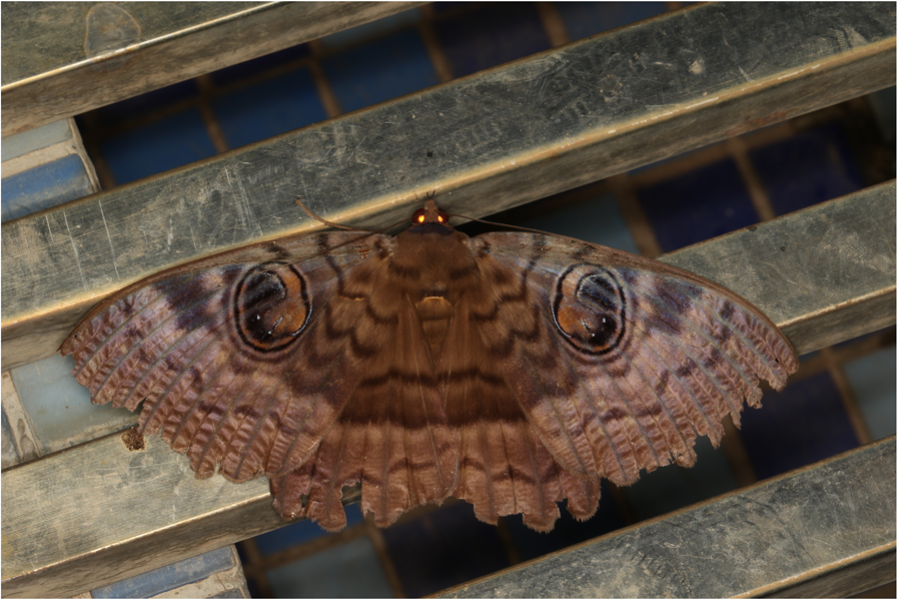
*Erebus walkeri* (Lep. Erebidae). Source: https://commons.wikimedia.org/wiki/File:Erebus_walkeri_(22115920295).jpg. Attribution: Donald Hobern from Copenhagen, Denmark [CC BY 2.0 (https://creativecommons.org/licenses/by/2.0)]

In Madagascar, many informants told me that there is a moth which terrifies the local population, because it is believed to embody the soul of an ancestor ([45]; p. 15). This is the night moth *Erebus* (*Nyctipao and Patula*) *walkeri* (Lep.: Erebidae) commonly called ‘lolo-paty’, meaning ‘spirit of the death’ ([46]; p. 82) and believe to bring misfortune [47]. The span of the black-grey wings is 7 cm and on the wings is a drawing of two black eyes (Fig. 3).

Lepidopterans’ fluttering flight with chops to the sides is considered to be a sign of madness by the Chokwe and Nkoya in Zambia ([7]; pp. 184–185). Insane or intoxicated persons who walk aimless are named butterflies/moths (also mentioned by the Lozi in Zambia). It is also thought that putting wing-powder of the butterfly/moth in food can make somebody mad and also that madness can be cured by rubbing it over the body. However, in Uganda (Ganda, Nyoro), it was believed that by touching the butterfly/moth you would get leprosy (referring to the scales) and (Nyoro) that you would get mad. In Cameroon (Bamilike), it was believed that lepidopterans could be ordered by someone to transmit diseases.

The seeing of lepidopterans is a sign that the rainy season is approaching (Kenya: Luo; Mali: Malinke; Tanzania: Iraqw, Digo; Togo: Ewe; Sudan: RES). It can also be a sign of a good harvest (Tanzania: Chaga, Sukuma), but what may be rotten (Benin: Fon). However, in Cameroon (Bamileke, Bani-Pahuin) and Uganda (Ganda, Nyoro), it announces the dry season and probably hunger.

In Togo, one informant (Kabye) told me that the scales of a lepidopteran are put on the pubic area of a child in order to give it the power of a man. Another respondent from that country (Akebu) specified that it was a yellow butterfly or moth, with the purpose to promote growth of hair in the pubic area. The practice was applied to 10–12 years old boys.

Concerning the African armyworm, caterpillar of the moth *Spodoptera exempta*, it is believed that the pest is associated with rain. The occurrence of many armyworms results in a good yield (Tanzania: Chaga; Zanaki; Sudan: RES; Togo: Ewe; Zanzibar). Also, when the crop survives, there will be a bumper harvest (Kenya: Kikuyu; Tanzania: Iraqw), even if they must plant again (Tanzania: Digo). This is a reason not to spray the crop with insecticides (Zanzibar). It is also believed that the armyworms drop from the sky; they come as rain (Tanzania: Chaga; Zanzibar). That is why the pest is also called ‘mystery worm’. Egg-laying of the adults is usually co-ordinated with the onset of local rains, so armyworm outbreaks coincide with the germination of new crops [48]. In Kenya (Kamba, Luo), Togo (Ewe) and Zimbabwe (Shona, Zezuru), I was told that armyworms are brought in by ancestors as a kind of punishment and insecticides should not be used. This was confirmed by an article in the Sunday mail of January 29, 1995:

In Chiweshe there is an outbreak of the armyworm. The local svikiros, a spirit medium of the Shona people of Zimbabwe, are the ones through which the ancestors talk and advices what to do get rid of the pest. They advised not to spray insecticides as it would violate the laws of the land and could lead to disaster. They asked the farmers to bring each three caterpillars and rituals were performed involving the brewing of millet beer and dancing. This because the pest is often considered to be a kind of punishment. The villagers have to be forgiven for what they did wrong. Both in the insecticide-sprayed crops and in the ritual-treated crops the pest disappeared. However, it happened that the insecticide-treated crops were invaded again and the people took this as a sign that they had violated the law of the country.

In Togo (Ewe), also rituals are performed to ease the ancestors. In Kenya (Kamba), with heavy infestation of armyworm in grasses, cows get swollen bellies and the only way is to satisfy ancestors by conducting ceremonies offering milk, honey and meat. Hobley [49] describes a ceremony in Kenya by a Kikuyu medicine man who was specialised in the removal of armyworms. He could get them killed by heavy rain, to be eaten by soldier ants or to be dried up by the sun. However, he could also bring a plague of caterpillars on people that treat him badly, by praying to the god Engai and by pouring beer in the village.

In Nigeria (Yoruba), a butterfly or moth entering a house during the day may be considered a good omen (e.g. a pregnant woman getting a safe delivery). However, during the night, it is a bad omen (Ebibio). In Cameroon (Bafia, Bakoka, Bamileke, Bassa, Yambassa), when a butterfly or moth enters the house, an important visitor is expected to come to the house. However, when it is a black one, there will be a death or in Kenya (Luo) it brings illness to the children. In Senegal (Serer) and Chad (Mbaye), the entering of a butterfly or moth is considered an ancestor, and therefore the animal should not be killed. In Africa, the ancestors, or the living-dead, are believed to be disembodied spirits of people, incarnated in animals such as birds, butterflies/moths, bees, snakes and lions [50].

In Cameroon (Bamilike), several informants told me about a caterpillar being ‘a protector of the unborn child’. Although it was mentioned that everybody knows it, I could not find a confirmation in the literature.

In Zambia, the Nkoya believe that the bagworm is female and that the male is a small snake person (7; pp. 166–172). The bagworms are considered dangerous because snakes are always in the neighbourhood. When treading upon a case and crushing it, the snake will attack and bite the person. In Zambia (Lozi), I was told that when bagworms are given to the husband in his food, he will remain loyal to his wife. In Kenya (Kamba), it was mentioned that when an uncircumcised boy loses cows, it is a serious matter and the boy then collects a bagworm and forces the caterpillar to come out. The direction to which it points when it comes out indicates that is where the lost cows can be found.

### Tales and proverbs

There are several proverbs dealing with butterflies/moths in use by the Yoruba (first two mentioned by my informants) [51].

- ‘Labalaba to digbo lu egun, aso re a faya’ (the lepidopteran that flies against thorns will have its cloth torn) which means: weigh your opposition carefully; otherwise, you may suffer enormous losses [52]. It resembles the next one.
- ′Yio b′ale, yio b′ale ni labalaba fi wo′gbo (it will settle down, it will settle down, yet still the lepidopteran flies into a bush thicket). There are several meanings: (1) ‘Looks and first impressions may be deceptive with respect to a person’s ability to get a task accomplished.’ [51]; (2) You try to catch the lepidopteran when it lands, but when you are close it flies away and finally you are lost in the forest. This incantation is used when you want to escape from your enemy; (3) Do not try to do the impossible. The wings of the lepidopteran may get damaged if it flies into the thorny trees.
- ‘Labalábá fi ara è wéye, kò lè ìse eye’ (the butterfly or moth likens itself to a bird, but it cannot do what a bird can do) meaning: attempts to emulate those better endowed and qualified than oneself prove futile ([53]; p. 75).

In Sudan (Dongolawi, Gaälien, Nubian), saying ‘he is like a lepidopteran’ means that somebody is a quarrelling type (a hard-headed person, difficult to persuade of something). The suicide of lepidopteran flying into the flame was mentioned in Nigeria (Yoruba). Such a proverb exists in Madagascar ‘Lolo fotsy mandoro tena ka main’ny nahim-pony’ meaning a white lepidopteran burns itself (at a candle) at his own will. It is about the evil that one seeks and gets ([39]; p. 146, [54]; p. 126).

The saying ‘this person is a lepidopteran ’ may also refer to persons being ugly, having bad manners or being slippery people (Rwanda: Toro; Chad: Wadai). This has to do with the spasmodic way lepidopterans fly (not straight).

The saying ‘Aza manantena landy latsaka ho lamba vita’ means do not expect that the fallen cocoons will become a silken cloth by themselves ([39]; p. 60, [54]; p. 91). Because in Madagascar the silkworm occupies an important role, silk as shroud and pupae as food, they are an example to people according to proverb ‘Ataovy toy ny landy; an-trano vao manatevina’, meaning ‘Do as the silkworm, in his house (cocoon) he keeps order’ ([39]; p. 61).

However for the Fon in Benin, a lepidopteran means liberty as expressed in the proverb ‘Awadakpεkpε wε un nyi’ which mean ‘I am a butterfly; I fly from flower to flower’ ([55]; p. 24).

In Kenia, the Kikuyu have a proverb ‘Arũme marĩ rũamba’ meaning ‘men have prickles like hairy caterpillars’ do not rub people the wrong way for you may get hurt (do not stir up a hornet’s nest) ([56]; p. 3).

In Nigeria (Yoruba), a proverb says ‘when a bagworm harvests sticks in the bush, he uses his head to carry them’ which means you must take responsibility for your actions [57].

### Art

The wings of a butterfly or moth are used for decoration (Nigeria: Yoruba; Uganda: Ganda): by gluing the wings on paper (Benin: Nagot; Togo: Ewe) or by pressing the wings between two papers which leaves the imprint of the wings on paper (Burkina Faso: Mossi) or by using the wings themselves (RCA: Gbaya). Also, the wings are used as a template for making design on cloths (Kenya: Kikuyu; Sudan: Gaälien, Kuku, RES). You also often see butterflies or moths depicted on stamps (Kenya: Kikuyu) [58].

**Fig. 4:**
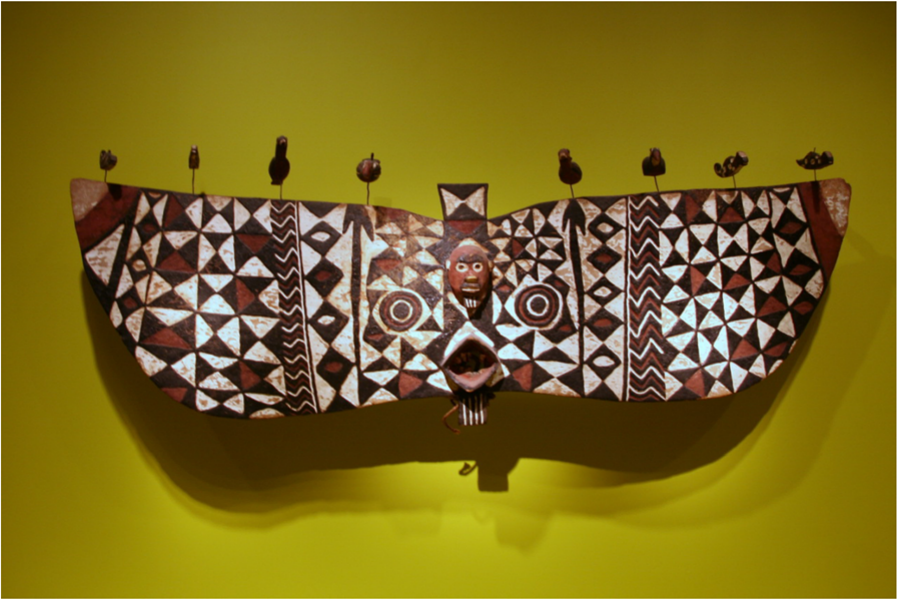
Butterfly mask Mask. Nuna peoples, Burkina Faso (National Museum of African Art). Source: https://commons.wikimedia.org/wiki/File:NunaMask.jpg. Attribution: cliff1066 [CC BY 2.0 (https://creativecommons.org/licenses/by/2.0)]

The masks from the Bwa in Dossi and the Nuna (subgroup of the Gurunsi) in Leo, Burkina Faso are made from wood of *Ceiba pentandra*. One of these masks is called butterfly masks (Fig. 4). The abstract mask consists of a broad, horizontal plank (about to 2.5 m long), decorated with large concentric patterns ([59]; p. 47). The mouth projects from the centre and the circles represent the patterns on the butterfly’s wings. Butterfly masks, called Yehoti in Boni (Department in the Province of Tuy in Burkina Faso), have eight enormous target patterns spread across their wings. The elders of the Kambi clan in Dossi claim that the plank masks represent flying spirits and are associated with water. These spirits can take the form of insects that mass around muddy pools after early rains. Plank mask are not representational, but embody supernatural forces that act on behalf of the Bwa clans that use the masks [60]; see also a video about these masks [61].

Sande or Bondo is a women’s secret society in Liberia, Sierra Leone, Guinea and the Ivory Coast. The Bondo society initiates girls into adulthood by rituals including female genital mutilation and prepares girls for marriage and motherhood. The society also instils notions of morality and proper sexual comportment and maintains an interest in the well-being of its members throughout their lives. The significance of the mask of the Bondo/Sande, called Nöwo (meaning butterfly/moth) by the Temne in Sierra Leone, is discussed by Lamp [62]. The mask is probably named after the chrysalis of a butterfly or vice versa. The masks and the chrysalis have a series of rings at the base (abdominal segments on the chrysalis). Around the back of the chrysalis, just at the top of the base rings, especially on the Danaidae (monarch butterflies), common in Sierra Leone, a horizontal row of small nodules (tubercle scars) may be seen. The large flaps on the sides of some masks seem to reproduce the wings of the chrysalis. Also, the antennae and legs seem to be reproduced on the mask. The chrysalis refers to the metamorphosis, just before adulthood, as is the Bondo initiation. According to ([63]; p. 134), the ontological theme of the initiation rite is metamorphic and psychologically and spiritually transformative. Females give up the behaviour of children and assume the roles and responsibilities of adult women in the community. Girls ritually die as children and are reborn as adults. The caterpillar of *Danaus chrysippus* (Lep.: Nymphalidae) the most common butterfly in Africa, feeds on toxic milkweeds, and the adult butterfly is itself so toxic that it is unpalatable to predator birds. Bondo is described as difficult medicine, preparing the women’s defences against the forces of evil. In addition, this butterfly has a life cycle roughly equivalent to that of the moon, and which is the unit of measurement used in the timing of initiation procedure.

**Fig. 5:**
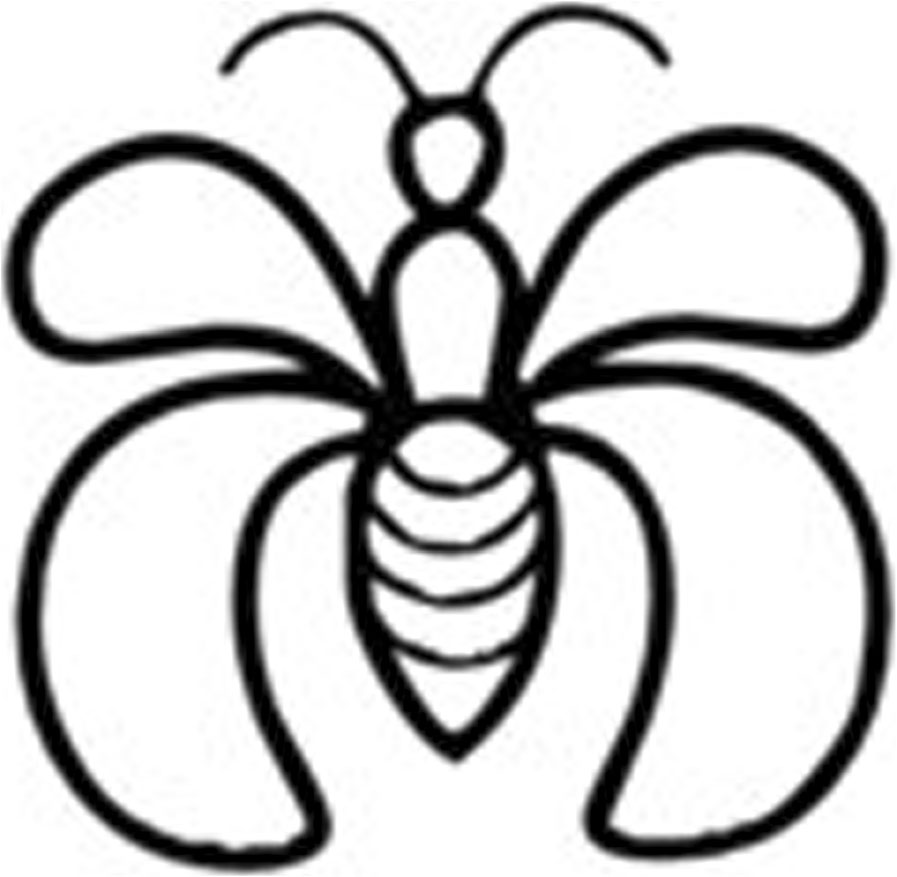
The Adranka butterfly symbol

The Ashanti in Ghana use in fabrics and pottery visual symbols called ‘Adranka’ that represent concepts or aphorisms. The Adinkra motif termed ‘Fafanto’ or ‘Esono Nantam’ is a butterfly symbol of tenderness, gentleness, honesty and fragileness (Fig. 5). The proverb ‘Fafanto se nsa ni o, na aka ho sika’ in Adranka means the butterfly may be fluttering around a pot of palm wine, but will not drink it; it cannot afford to buy it ([64]; p. 5).

In Kenya (Kamba), a string of dead butterflies as necklace or headband is used for decoration and also during circumcision ceremonies. Children tie ropes on butterflies/moths to play with them (Tanzania: Chaga). They use the fine threats from de pseudo stems of banana or from the leaves of the sisal plant.

The author visited ‘Reserve Peyrieras’ in Madagascar where non-mammalian animals are reared for study, captive breeding and export. These include butterflies and moths such as (1) the comet moth or Madagascan moon moth (*Argema mittrei*; Lep.: Saturniidae) of which the male has a wingspan of 15 cm and a tail span of 20 cm, making it one of the world’s largest silk moths; and (2) the Madagascan sunset moth (*Chrysiridia rhipheus*; Lep.: Uraniidae) considered one of the most impressive and attractive lepidopterans; (3) the swallow tail butterfly *Papilo delalandei* (Lep.: Papilionidae), arguable the most beautiful in Madagascar. In Madagascar, there are about 300 species of Lepidoptera of which more than 70% endemic [65].

### Literature

Tango Muyay ([18]; pp. 15–113) describes 33 ethnospecies of edible caterpillars mentioned by the Yansi and Mbala in Bandundu, a province in the western part of Democratic Republic of Congo. For most of the species, the following information is given: a description, how they behave, how they are collected, how they are prepared and then stories, proverbs, songs, taboos and medical properties related to them. One song will be given about a caterpillar considered to be very delicious called ‘Miliem’. This mature caterpillar comes down from the tree (very likely in order to pupate in the soil) at about 16:00 h. Women must wait and see whether they come down and when they do, they are collected. The women often get back very late in the night because of the harvesting. One of the songs related to this caterpillar goes as follows:

Miliem caterpillar come down quick, because there is a woman with childbirth pain.

Come down quickly because she wants to go back to the village for the delivery.

If you do not come down, she will deliver here in the forest.

The caterpillar could be *Imbrasia epimethea* (Lep.: Saturniidae), as it descends from the tree to pupate.

In Masai, the story ‘Enkatini e Olkurto o Olikai Ilcang′it’ (the caterpillar and the wild animals’) ([66]; 184–185) goes as follows.

While hare is from home, the caterpillar takes possession of the home. When hare returns home and seeing the tracks, he cries out ‘who is in my house?’ The caterpillar responds ‘I am the warrior of the long one whose anklets have become unfastened and in the fight in the Kurticele country, I crush the rhinoceros to the earth and make the cow’s dung of the elephants! I am invincible!’. Scared hare leaves and brings successively the jackal, the leopard, the rhinoceros and the elephant, all of whom decline the challenge when hearing the caterpillar’s boast. When hare brings the frog, he answers after the caterpillar’s boast ‘I, who am strong and a leaper, have come. My buttocks are like the post and God made me vile’. The caterpillar trembles and answers, ‘I am only a caterpillar’ and all wild animals laugh at him and drag him out. In this case a boast is being responded with a counter boast.

The lesson seems to be that self-exploits may be rewarded.

‘Butterfly burning’ is a novel by Zimbabwean writer Yvonne Vera, first published on January 1, 1998 [67]. Set in the late 1940s, it is about the voice of the people under colonialism in Zimbabwe. The construction worker Fumbatha falls in love with Phephelaphi, a much younger woman, who soon tires of his devotion. She would like to become self-sufficient, but after gaining a position at a neighbourhood nursing school, she learns she is pregnant and no longer qualified for the class. Therefore, she induces an abortion to be able to apply for the program. Questioning his love for her, he ends up cheating on her. At some point, he later admits to an affair with another woman. At the end she sets herself on fire as Fumbatha enters their house. The novel was ranked as one of Africa’s 100 best books of the twentieth century.

Another book ‘Butterflies over Africa: perspectives on changing and transforming the continent’ gives practical thoughts and guidance on new ways to approach human development and understand leadership in African contexts [68]. It proposes a holistic way of re-conceiving development, as a process of transforming lives.

### Dance

The Zu/′hoasi are a !Kung-speaking San group living in the western Kalahari on the Namibia-Botswana border [69]. Zu/'hoasi women engage in a caterpillar dance escorting a young woman to the cleansing hut as she approaches menses. She will wait there until her first period is over when the women ritually clean her. On YouTube, a video can be watched of this caterpillar dance [70]. Seeing how the women move in line, it is clear why it is called caterpillar dance.

From the DRC, I was also informed that ‘Soukous’ (from French ‘secouer’, ‘to shake’) is a popular genre of dance music from the Congo Basin. It derived from Congolese rumba in the 1960s and gained popularity in the 1980s in France. If you take a caterpillar by the head you get a similar movement as the dancers make [71].

Peigler [72] discusses the use of ankle rattlers made from cocoons of the Saturniidiae family (Lepidoptera). It seems to occur mainly in southern Africa (Botswana, South Africa, Namibia, Zimbabwe), and is in use among the following ethnic groups: San among which: !Kong, Sotho, Venda and Zulu. The cocoons are from different species of Saturniidae [(*Argema mimosa*, *Epiphora* sp., *Gonimbrasa postica*, *Heniocha* sp. by a San informant from South Africa] and of Lasiocampidae (*Gonometa rufobrunnea*) and sown onto skins of goat, kudu and monkey. The cocoons are filled with small stones, broken sea shells or with ostrich shells. The fur is on the inner side. The cocoons are sown in rows (the author got rattles with three rows of 12 cocoons) on the outside of the skin (fur on the inside). You can watch a dance with rattles from the Zulu on YouTube [73].

## Conclusions

Lepidoptera are among the most beautiful insects in the world and because of their aesthetic value constitute a source of inspiration in art. They also encourage eco-tourism as was shown by an insect farm in Madagascar. Lepidoptera provide ecological services such as pollination, while they also serve as food for birds, spiders and lizards, although this was not mentioned by my informants. However, the consumption of caterpillars by humans was very often mentioned and many species are eaten in central Africa. Lepidoptera are also considered as symbols of souls and freedom. Their transformation from one form to the other, the metamorphosis, is a symbol for the central event in female puberty, the menarche. The element of drastic change has also been used in African literature as a metaphor for political change.

The relation of lepidopterans with religion is shown in several ways. Names of butterflies/moths in the mainly Muslim countries of the Sahel region refer either to God or to their religious leaders. The association of black butterflies/moths with the soul of the dead (ancestors) was mentioned in Chad, Senegal and Madagascar. In the last country, this was for *Erebus walkeri* which is a black moth and has eyes on the wings. In Greek mythology, Erebus (also Erebos) was conceived as a primordial deity, representing the personification of darkness. In Greek literature, the name Erebus is also used as a region of the Greek underworld where the dead pass immediately after dying [74]. So, the name is very appropriate.

The metamorphosis is often not known and when known, people are ignorant of which butterfly or moth derives from which caterpillar. This probably is not true for wild silkworms, because they are often semi-domesticated. In some countries in West Africa (Liberia, Guinea, the Republic of Côte d’Ivoire and Sierra Leone), the Bondo initiation is performed; the genital mutilation prepares girls for marriage and motherhood, symbolising the transformation from girls into adulthood. The mask used represents a pupa. Lepidoptera universally exemplify transformation and change and for this reason some books have titles such as ‘Butterflies over Africa’ by Assegid [68].

The behaviour and morphology of butterflies/moths also appeals to the imagination. For example, the erratic way lepidopterans fly refers to a slippery person, or the way they flap with their wings, like the up and down movement of a Muslim praying. Also, dances have been derived from the way a caterpillar moves. The most striking is the way a bagworm envelops itself with sticks, giving it the name of collector of firewood in Cameroon. It can be used as magical concoction to make sure a husband stays home and remains faithful to his wife, like the bagworm is housed. Moreover, it is used as a medicine for several complaints. Reference is also made in Zambia to the isolation by menstruating girls in grass-covered shelters.

There are also products derived from lepidopterans and the most used is that of silk. When the silk is not from the domesticated silkworm *B*. *mori*, then they are called wild silks often collected from nature. These products are often much more expensive than the common silk. Therefore, it has been suggested that these resources should be better exploited in a sustainable manner as they are currently threatened by deforestation and overexploitation [75]. The same author suggests ways for a better exploitation [43]. In Madagascar, wild silks are very expensive and they are used in burial ceremonies. Silk from *Anaphe* spp. are very expensive and only used for special ceremonies. The *A*. *panda* silk nest is even used as purse. Apart from silk the cocoons may be used as ankle rattlers in dances.

When plagues occur, such as armyworms in East Africa, it is often believed that they were sent by ancestors because the community misbehaved. Offerings must be made to in order to satisfy the ancestors. Ancestors are often incarnated in animals, among which lepidopterans [50].

The appearance of lepidopterans is either a sign of the dry or the rainy season. That is because lepidopterans can survive the dry season as pupae/chrysalis or eggs. When as pupae/chrysalis then the butterflies or moths appear at the beginning of the rainy season. However, when they pass the dry season as eggs, then they start their development on plants in the rainy season and at the end of it, they pupate and appear as butterflies or moths in order to oviposit their aestivating eggs.

The behaviour and morphology of butterflies/moths and caterpillars provided the inspiration for proverbs cultures in many African cultures. This may have to do with the way the lepidopterans fly or the irritating hairs of the caterpillar.

It is also noteworthy that some beliefs are really confined to one country, for example calling the bagworm a collector of firewood (Cameroon). Others are widespread such as calling the lepidopteran a Muslim spiritual leader in the Sahel. Products from lepidopterans as human food and silk are very much appreciated in Africa. Moreover, the insects are used in medicine, art and serve as symbols in religion, proverbs and predictor of events. The most striking feature of metamorphosis is often not recognised by common people.

In this article, no reference was made to how other cultures in the world deal with symbolism of lepidoptera, e.g. in western art [76]. However, when looking at the characteristics summed up, they resemble many issues mentioned in this article. To mention just a few: the spasmodic flight, ugliness, death, self-destruction, the reincarnation and the change of seasons. It is remarkable that this insect group in sub-Saharan Africa is so important as a provider of products and as an inspiration for many aspects in the culture of human life.

## Abbreviations

CAR: Central African Republic
DRC: Democratic Republic of Congo
Lep.: Lepidoptera
RES: Resource persons
Sp(p): Species

## Author’s information

Arnold van Huis is emeritus professor of Wageningen University & Research in the Netherlands. He worked as integrated pest management specialist for the Food and Agriculture Organisation of the United Nations from 1974 to 1979 in Nicaragua, analysed and published the data collected in this country from 1997 to 1982 at Wageningen University, was leader of a Sahelian crop protection training project for the Netherlands’ Ministry of Foreign Affairs from 1982 to 1985 in Niger, and worked afterwards as tropical entomologist at Wageningen University, the Netherlands. He is now fully engaged in the topic of insects as food and feed.
